# Low-Cost Device for Measuring Wastewater Flow Rate in Open Channels

**DOI:** 10.3390/s24206607

**Published:** 2024-10-14

**Authors:** Daria Wotzka, Dariusz Zmarzły

**Affiliations:** Faculty of Electrical Engineering, Automatic Control and Informatics, Opole University of Technology, 45-758 Opole, Poland; d.zmarzly@po.edu.pl

**Keywords:** low-cost device, flow rate determination, ultrasound velocity measurement, wastewater level measurement, open channel, cross-correlation

## Abstract

This research paper describes the development of a low-cost device for measuring wastewater flow rates in open channels, a significant advancement enabled by the evolution of microcomputers and processing techniques. A laboratory setup was constructed to validate the device’s accuracy against a standard flow measurement method, optimizing key parameters to achieve a linear relationship between detected and set flow rates, while considering hardware limitations and energy efficiency. The central focus of the research was developing a method to measure the velocity of contaminated fluid using ultrasonic signals, employing the cross-correlation method for signal delay analysis in a stochastic environment. This was complemented by a procedure to measure fluid levels, also based on ultrasonic signals. The device’s reliability was assessed through repeatability and uncertainty measurements, confirming its accuracy with an extended uncertainty not exceeding an average of 3.47% for flows above 40 L/min. The device has potential to provide valuable data on the operational dynamics of sanitary networks, crucial for developing and calibrating simulation models.

## 1. Introduction

The development of cities and villages, driven by the steady increase in population, results in an increased volume of wastewater flowing into the central sanitary sewer infrastructure. While the construction of new residential areas is associated with the simultaneous construction of sanitary sewers of appropriately adjusted capacity, in some urban areas, there is no physical or economic possibility to expand the existing, often open, channels that currently frequently operate at the limit of their capacity.

Improving the monitoring system of sanitary networks is currently a significant branch of the industry, enabling the assessment of their hydraulic efficiency. The main problem faced by water and sewerage companies concerns the systematic collection of data, including the volume and velocity of wastewater flow in open channels and the thickness of the sewer sediment in man-entry collectors. A lack of supervision over the proper functioning of the sewage discharge system can lead to system overflows or leaks, posing a potential threat to the life and health of the region’s residents and causing environmental contamination. Access to current operational parameters of the system allows for the estimation of the load in specific areas and the detection of undesirable phenomena, such as surges and backups in the channel or exceeding maximum fill levels.

One area of current scientific research involves constructing simulation models of sanitary systems, which can be used to predict network behavior under various working conditions. The values of the operational parameters of the sewage system, which are recorded in real time, constitute a significant and necessary element in the process of calibration and validation of mathematical models. The phenomenon of fluid flow in sewer channels has been described to some extent in the literature. Equations and mathematical models characterizing flow through channels of different shapes, such as trapezoid, rectangle, or cylinder, have been identified [1,2,3,4,5]. However, sewage systems, especially in large agglomerations, are diverse, composed of channels of various sizes and shapes with numerous intersection points.

Currently, many water and sewerage companies still do not have metering systems for sewage or stormwater infrastructure. Measuring the linear velocity of flow in successive sections of the network remains a technological challenge and a significant aspect of the development of existing sewer infrastructure. The issue arises from the necessity of installing measurement devices, usually in highly contaminated channels, in a possibly non-invasive manner that does not disrupt the proper flow of sewage. Moreover, measuring the actual amount of wastewater discharged can result in significant savings for plants that use water in their technological processes. Presently, in manufacturing plants, sewage is accounted for based on the amount of water drawn or on a flat-rate basis. In reality, for industrial installations, the amount of wastewater discharged into the sewerage may differ, e.g., due to water losses in production processes or due to losses in the exploited installation. This is an important reason why it is worthwhile to expand existing systems with additional monitoring devices. Although there are currently several solutions on the market that allow for the measurement of flow velocity in open channels, these options tend to be relatively expensive, and their measurement accuracy, along with the need for calibration, indicates a continued need for the development of new methods and devices with minimized construction and operational costs.

The innovative use of ultrasonic signals to measure fluid velocity represents a significant leap forward in the accuracy and reliability of flow measurement in contaminated environments. By employing the cross-correlation method for signal delay analysis, this research not only enhances the precision of flow rate determinations but also provides a viable solution for environments that present unique challenges, such as high levels of turbulence and contamination. Our work not only contributes to the field of environmental monitoring but also supports broader goals of sustainability and public health by enabling more effective wastewater management strategies.

Later in the article, in Section 2, we provide a comprehensive literature review of current advancements in ultrasonography for flow measurement across various industrial sectors, highlighting its diverse applications and emerging trends. In Section 3, we focus on the cross-correlation method, which is crucial in flow rate measurements, and we present the detailed process of designing and constructing a specialized low-cost device aimed at measuring wastewater flow rate in open channels, highlighting the challenges and innovative solutions involved in its development. Section 4 describes example measurement results of data from the velocity and level sensors and validation of the device’s performance under laboratory conditions. Section 5 presents a discussion of the results achieved, while Section 6 concludes the paper.

## 2. A Review of Ultrasonography in Industrial Flow Measurement: Applications and Current Trends

Ultrasonics have found widespread application in various industrial areas, such as ultrasonic distance measurements [6], ultrasonic non-destructive testing [7], and ultrasonic flow meters [8], due to their good properties, including high penetration and information transfer capability [9]. The fundamental advantages of the ultrasonography technique include the non-invasiveness of the flow and a high dynamic range [10]. Currently, there are many industrial applications where various methods of liquid flow measurement are used. This includes systems for sludge transport, water supply for irrigation systems, and river flow control [11], where accurate measurements are crucial for safe operation, including proper flow distribution and control. In open channels, the flow is usually difficult to measure directly. Most methods rely on calculating flow rate based on measurements of other variables that can be directly measured. These variables include channel width, depth, slope, and flow velocity. The most commonly used methods for measuring flow in open channels include the following [12]: time-gravimetric method, tracer-dilution method, area-velocity method, hydraulic radius slope method using the Manning’s equation (Gauckler–Manning–Strickler formula), and methods using hydraulic structures, such as weirs or flumes.

The time-gravimetric method is limited to flow rates less than 100 L/min and is not suitable for calculating continuous flows. The tracer-dilution method involves adding a certain amount of concentrated dye directly to the liquid. To determine the degree of dilution of the evenly mixed concentrate at a certain distance from the point of discharge, chemical analyses are used. A constant, known flow rate is a condition for obtaining reliable results. The advantage of this method is that measurements of the channel geometry in which the flow is being studied are not required. Markers can be diluted with dye or salt, where the former is suitable for measuring both small and large flows (due to the relatively low cost of the dye), and the latter is dedicated to turbulent streams, where other flow measurement methods are not practically applicable.

In the hydraulic radius slope method, Manning’s equation is used as a model of flow resistance. This method is used to measure uniform flows in open channels. For flow control purposes, the hydraulic radius slope method is not recommended due to a large measurement error in the range of 25–30%. This is caused by uncertainty in determining the correct friction parameter, i.e., the Manning roughness coefficient, which characterizes the flow. Another commonly used method involves the use of features of hydraulic structures, such as weirs and flumes, which introduce restrictions in the direction of flow, leading to changes in the values of velocity and depth of the liquid in the channel. The measurement of flow intensity in the case of a channel or weir is based on measuring the depth of flow established by the applied structure. Although, within laboratory observations, measurements for flumes and weirs show high accuracy, with errors of about 2–6%, the inaccuracy of online (field) measurements still falls within the range of ±10% [13]. This is due to the uncertainty of level measurement, as well as the difficulty in obtaining the correct discharge coefficient for the correction of losses in the theoretical depth–flow relationship. Efforts are underway to improve this method.

In the area-velocity method, measurements of average flow velocity, V, through a known cross-sectional area, A, are used. In this method, a pressure transducer and a Doppler ultrasonic sensor are used to measure depth and velocity, respectively. These instruments are sensitive to flow disturbances, which results in a measurement error of approximately ±10% [12]. In this area, two methods of measuring fluid velocity are used: the Doppler method and the correlation method [14], which will be described later in the paper. The ultrasonic velocity profiler (UVP) method was introduced by Takeda for determining blood velocity profiles for medical applications [15]. This technique allows for the acquisition of information about the spatial distribution of flow. Takeda found that the accuracy of the measurement and the spatial and temporal resolution are closely dependent on the frequency of the ultrasonic signal, the structure (shape) of the pulse, and the parameters of the electronic equipment used in the studies. The described method is limited by physical phenomena associated with the attenuation of ultrasonic signals; hence the propagation occurs only to a certain depth.

In the UVP method, it is assumed that the fluid contains a certain number of particles that reflect the signal but can also disturb its flow or change its fundamental parameters [15]. In the work [16], the application of different flow measurement methods was considered. In particular, the cross-correlation method, an advanced technique of ultrasonic Doppler (USD), was presented for measuring velocity and flow rate in liquids and gases. To determine the flow rate in sewage, continuous-mode acoustic sources and independent measuring sensors were used. To improve measurement accuracy, the pulse Doppler (PD) technique was applied. This technique allows for determining the velocity profile with a spatial resolution of several decimeters and is suitable for measurements in large areas, such as oceanography. According to the authors, the cross-correlation technique has significantly greater accuracy in determining velocity, as well as spatial resolution, resulting in an accurate estimation of the flow profile. This method can also be used for dimensions with a spatial resolution of 1–2 cm. In larger objects, the coded pulse method, also known as chirping, can be applied. This coding method is also used, for example, for modulating signals in modern 4G and 5G data transmission technologies. For this reason, the chirping technique is applied when high accuracy of flow measurement in an open channel is required [16].

In [17], the authors compare acoustic Doppler velocimetry (ADV) and particle image velocimetry (PIV) in measuring open channel flow. They find notable differences in their performances, particularly in measuring time-averaged velocity, turbulence, and other parameters. ADV’s effectiveness is limited to a small “sweet point” within its profiling range, beyond which it struggles to accurately quantify turbulent parameters. It highlighted the need for careful evaluation of ADV measurements to accurately describe turbulence in open channel flows.

In [18], the characteristics of a developing narrow open-channel flow were analyzed using ADV and laser Doppler anemometry (LDA). The study aimed to (1) characterize flow using LDA; (2) assess the intrusion effect of ADV sensor immersion; and (3) compare ADV and LDA measurements. Findings included high turbulence in outer flow regions and flow profile changes due to ADV sensor intrusion, varying with the Froude number. ADV was noted to underestimate flow and turbulence compared to LDA, which was attributed to its lower sampling rate and potential intrinsic noise. The practical implications of these findings were also discussed.

In the work [19], a single resonant frequency UVP method was described, which was developed to measure the instantaneous velocity profile in two-phase flow of liquid and air bubbles. A single-channel resonant frequency transducer, single-channel pulser-receiver, and microprocessor for data processing were used. A modified Doppler frequency decomposition (DFD) technique was applied, which allowed for classifying the Doppler frequencies of particles (liquid phase) and bubbles (gas phase). This enabled the authors to determine the velocity profile for both the liquid and the bubbles. The experiment was conducted in a vertical pipe flow system. The measurement technique described in [19] works in clean water with air bubbles, but when the fluid contains many particles of different sizes and shapes, the described method is not applicable, as the signals reflected from individual particles overlap in time. The UVP system for studying flow characteristics in a spatio-temporal form was also presented in article [20]. UVP is currently used in a wide range of flow configurations, not only in limited geometries such as flow in pipes or channels [21,22,23] but also in naturally occurring open channels, such as rivers or lakes [24]. In article [24], issues specific to independent ultrasonic instrumentation dedicated to sewage systems were considered, allowing for online monitoring of water height, velocity profile, and granulometric size class distribution of the suspension. The instrument described in the cited article enabled flow measurement with a 5% inaccuracy in open channels with diameters from 0.3 to 1 m and velocities up to 3 m/s, as well as measurement of suspension concentration with granulometric estimation. It is also applicable to opaque liquids, such as liquid metals [25] or chemical agents [26]. In article [27], a non-Newtonian fluid was tested in two different complex geometries using the UVP method. Velocity profiles were measured at three different positions in the center of a specially produced diaphragm valve with a 50% open space. The complex coordinates of the geometry and the magnitude of the velocities were analyzed and compared with the volumetric flow measured using an electromagnetic flow meter. The difference between the calculated and measured flow ranged from 15% to 25%. It was found that the most important issue for increasing measurement accuracy is the estimation of the location of wall connections, resulting from the close field of view of the ultrasonic transducer. This problem can be eliminated by introducing a next-generation transducer with improved parameters.

Simultaneous measurements of liquid velocity and contact profiles for layered smooth and wavy flows in a horizontal channel was performed in the work [28] using a UVP meter. The influence of ultrasonic pulse reflection on the gas-liquid interface and the bottom of the channel was reduced by applying an absorbent for ultrasonic pulses on the bottom wall and optimizing the level of the liquid and the time interval between pulses. For comparative purposes, rotational velocity measurement was performed using the particle-tracking velocimetry (PTV) method by taking video images simultaneously with UVP measurements. Polystyrene balls were used as reflectors and markers for both UVP and PTV measurements. The velocity profiles measured for wavy flow with periodically generated interfacial waves were consistent with the theoretical prediction for solitary waves. The turbulent component appeared in the velocity profiles for both smooth and wavy flows.

One of the key techniques in ultrasonographic applications is the measurement of the time of flight (TOF) of the ultrasonic signal from the transmitter to the receiver. The accuracy of determining the TOF values is currently being intensively explored in a number of scientific works [9]. For example, in article [6], a system is presented that performs accurate, non-contact measurements of the distance traversed by ultrasonic waves in air using a simple system design. The authors of work [29] consider issues related to determining the distance between the transmitter and receiver using ultrasound. They presented a combination of two distance-measuring principles, namely TOF and phase shift (PS). The properties of sinusoidal signals were used to determine the phase shift (PS) between the transmitted and received signals with relatively high accuracy. The problem of correctly estimating the total number of wavelengths, due to the uncertainty of the TOF method, was minimized by combining information from the TOF and PS parameters. It was confirmed that the combination of both methods significantly improves the accuracy and precision of distance calculations.

The authors of paper [30] analyzed the characteristics of the ultrasonic echo to find the appropriate characteristic point for calculating the propagation time of the ultrasonic wave. The characteristic point is necessary to maintain stability at the same gas flow intensity and changes with the propagation time of the ultrasonic wave, which may reflect the pattern of propagation time variability with gas flow intensity. Based on the similarity characteristics of the normalized ultrasonic echo signal, the authors proposed a signal processing method based on a variable threshold ratio and zero detection to eliminate problems arising from changes in the amplitude of the ultrasonic echo signal and to increase the anti-interference capability of the threshold method. Their method was implemented in a device for real-time digital signal processing, consisting of an FPGA system and a DSP processor.

Calibration experiments on gas flow were conducted to verify the effectiveness of the proposed method and the proper operation of the developed device [30]. Digital processing techniques for ultrasonic signals to determine the distance between the transmitter and receiver are also considered by the authors of paper [31]. The low transmission bandwidth of ultrasonic transducers for range measurements in air causes the generation of waves with relatively long rise and fall times. This causes errors in situations where echoes are detected using simple techniques, such as threshold-crossing. In article [32], signal processing methods used in an ultrasonic gas flowmeter with high variability using TOL were presented. A method of emitting a sequence of pulses and an autocorrelation method was used. Accurate estimation of TOF values is a challenging issue in the field of ultrasonic flow measurement. High temporal resolution is required, especially in a measurement environment with a small internal diameter and a short sound path, as the speed of sound in a liquid is quite high, and the order of magnitude is in nanoseconds.

A newer method of obtaining differential time of flight is the spline-based algorithm, proposed in work [33]. The computational accuracy of this algorithm is higher than that of the cross-correlation algorithm, which is why the authors of work [33] proposed an improved spline-based algorithm to reduce computational costs while maintaining accuracy. Additionally, the mounting location of the transducers is one of the most important factors affecting the accuracy of the ultrasonic flowmeter measurement. The authors of paper [33] also analyzed the sensitivity of the separation distance between transducers. The result of the experiments showed that the improved algorithm is effective, and the separation distance has a significant impact on the result.

In recent years, there has been a rapid increase in the use of ultrasonic arrays for non-destructive evaluation (NDE). Article [7] presents a review of work on such arrays for applications in NDE. It also describes work related to their use in medicine and sonars. Summarizing the review of scientific publications, the authors concluded that although there are many common foundations, the use of arrays in NDE poses clearly different challenges for individual applications. An ultrasonic array consists of a single transducer containing a series of individually connected elements. Arrays offer great potential in improving the quality of measurements and shortening their execution times. Their main advantage over traditional single-element transducers is increased flexibility, meaning that one array can be used to perform many different examinations. Another significant advantage is the ability to instantly create images of the examined structure. These advantages have led to the rapid adoption of arrays by industry, as evidenced by a wide range of published research describing new piezoelectric materials, array geometries, modeling methods, and testing approaches [7].

In metrology, measurement uncertainty is a crucial issue. The accuracy of flow measurement stems from the fact that flowmeters are usually calibrated under ideal conditions, such as a completely defined velocity profile and high stability of the tested liquid. On the other hand, actual flow conditions are usually very different from the ideal. The flow profile is generally not fully developed but is often distorted or swirling. The temperature and pressure of the liquid can fluctuate significantly, and most conventional flowmeters are strongly dependent on these factors. This means that the accuracy of a flowmeter calibrated under ideal conditions is not guaranteed under actual conditions [34]. Article [34] describes the results of an analysis of the uncertainty of flow rate measurement in a pipe using the ultrasonic Doppler method and an assessment of the estimated uncertainty through actual flow calibration. The uncertainty was estimated for internal factors from the UVP measurement devices and external factors dependent on on-site measurements, such as the angle of inclination of the ultrasonic transducer. The relative uncertainty caused by internal factors was estimated to be 0.34%. The external relative uncertainty, considering external factors, was estimated to be between 0.42% and 2.13% depending on the angle of inclination of the transducer. The results of the actual flow calibration under the same conditions as the uncertainty analysis fall within the range of uncertainty considering internal factors.

This issue was also considered in article [35], where the measurement uncertainty of the ultrasonic beam emitted by immersion transducers in non-destructive testing applications was evaluated. The authors of [36] introduce an algorithm to enhance flow rate measurement accuracy in open channels, addressing challenges due to varying fluid properties and flow conditions. The algorithm adjusts flow rate models by comparing actual fluid depth with simulations based on the Saint Venant equations. Significant improvements in accuracy are reported, notably a reduction in error from ±2.3% to ±0.8% using the Venturi flume method. The approach also shows promise for accurately computing flow rates in unsteady-state flows across a broad range of conditions. The uncertainty of the ultrasonic beam parameters from immersion probes for non-destructive testing was assessed using Monte Carlo simulations and the Guide to the Expression of Uncertainty in Measurement (GUM). Calculated parameters, such as distance, focal length, focal width, and beam divergence, were determined according to EN 12668 2 [37]. Monte Carlo simulations were conducted using another program that generates pseudo-random samples for the distributions of the quantities under study. In all cases, significant statistical differences were found between the Monte Carlo and GUM methods [35].

In work [38], the impact of the content of water dissolved in crude oil was evaluated, and measurement uncertainty was investigated using ultrasonic flowmeters, considering that the total uncertainty of the measurement system should not exceed 0.3%. Work [39] aimed to improve the method of calculating velocity in liquids with a low level of acoustic scattering by applying a technique to solve the problem by seeding the flow with hydrogen microbubbles generated through electrolysis. In particular, the impact of the density of gas bubbles on the quality of velocity profiles based on the Doppler method in open-channel flow was examined. Bubbles were generated during the electrolysis of water using various electric current values. In all measurement techniques, the goal is to increase accuracy and precision. In Doppler ultrasonic telemetry, these features largely depend on the Doppler signal-to-noise ratio and the operation of the velocity estimator.

The most commonly used estimation method in coherent UDV is the pair of pulses method. Its success stems from the computational efficiency of the applied algorithm. However, for a wide range of experimental flows, pair pulse estimation is less efficient, especially when the liquid is pure water or concentrated sludge, where the signal-to-noise ratio can be very low, or in highly turbulent flows, where the Doppler signal has a wide spectrum [40]. The method presented by the authors of paper [40] is based on processing Doppler spectral information. It utilizes parametric identification inspired by theoretical models and experimental observations. The authors of the cited work developed a fast velocity estimation algorithm that surpasses the accuracy of the pair of pulses method. The effectiveness of the method was assessed by adding different levels of Gaussian white noise to the experimental Doppler signal.

In [41], the authors evaluate the accuracy of Parshall flumes used for measuring wastewater flow in Minneapolis. The study combines numerical studies using large-eddy simulation (LES) and level-set methods for turbulent, two-phase flow with field measurements using dye dilution and on-site tools. The research aims to determine the error margin of these flumes and water surface fluctuations, with LES and level-set methods providing insights into water surface variations and flow rate measurement uncertainties. Paper [42] focuses on enhancing river velocity and discharge measurement techniques. The study introduces a sub-pixel correction technique within a convolutional neural network (CNN) framework, marking a novel application of CNNs in image-based velocity measurement. Benchmarks include various flow models with synthetic noise interference. CNN outperforms DCC, particularly under noisy conditions, showing lower error rates. Real-world testing in a flume demonstrates CNN’s superior performance and potential for field application in water resource management.

Another area of focus for research groups worldwide is the development of new devices for flow measurement. Ultrasonic flowmeters are considered one of the fastest-growing techniques in the general group of devices for monitoring, measuring, and controlling processes [8,10,43]. They are a key element of ultrasonic technology. Ultrasonic flowmeters are used for liquids, gases, and multiphase mixtures, albeit not without limitations. No single technique or type of interaction within a given technique can be suitable for all types of liquids, problems, and situations. The choice of ultrasonic flowmeter type depends on several factors, including whether the flow is single-phase or multiphase, ease of use, type of flow (laminar, transitional, or turbulent), type of mounting (invasiveness), accuracy class, response speed, and reliability independent of temperature conditions, price, and maintenance and operation costs.

The topic of Coriolis flowmeters was considered by the authors of paper [44]. Measurements made using them depend on a constant velocity distribution within their interior, as certain features of the liquid’s vibration field are not uniform inside the measurement tube. This dependence is confirmed by the results of simulations of two simple tube configurations: one operating in beam mode and the other in shell mode. Further work on flow measurement in open channels, including non-stationary ones, is discussed in paper [45]. In article [46], an attempt was made to estimate the flow rate of drilling mud using a Venturi flow channel model. The study showed that the required tuning parameter of the model depends on the fluid’s properties due to its non-Newtonian behavior [47]. In work [48], an ultrasonic velocity profile measurement system was presented that is capable of measuring velocities for very slow flows. This system utilizes a new phase difference method, allowing for application in low-speed ranges, which poses limitations for conventional signal processing algorithms in the Doppler method.

Based on the combination of ultrasonic and Coriolis flow meters, article [49] proposed a method for measuring individual, mass two-phase gas-liquid flow with low liquid loading. In work [50], a flow meter was described for use in the Inolivent-4 total ventilator prototype for measuring unstable flows. The study [51] presented a constricted-type flow meter with an optimized flow profile for accurate measurements of liquid hydrogen flow. This work employed numerical simulation and multidimensional, multi-objective optimization to minimize the flow meter’s loss coefficient and the required length of installation. The article [52] reported experimental work on developing a clamp-on flow meter for measuring small fluid flows in pipes of small diameter.

## 3. Materials and Methods

### 3.1. Cross-Correlation Method

In the research presented in this paper, a flow measurement method based on the work of O. Binnefous and P. Pesque [53] was applied. This method uses cross-correlation analysis, which relies on the time shift between correlated successive echo signals rather than the frequency shift characteristic of traditional Doppler techniques. This method originates from the field of medicine and was used to measure blood velocity in living organisms by Teufel [54].

In measurement devices using the Doppler pulse method, a high degree of correlation between successive ultrasonic emissions is assumed. Algorithms developed to extract information about the velocity of fluids in channels are based on this correlation [55]. Correlation, a mathematical method of assessment, has found its place in flow measurement technology assessment, thanks to the development of fast and efficient microprocessors. In various flow measurement methods, it is particularly used as a non-invasive technique in multiphase flows. Combined with ultrasonic technology, it can be used in fully or partially filled large-diameter pipes, as well as in open channels. The prerequisite for using correlation is the presence in the propagation medium of particles that reflect the wave, floating solid particles or gas bubbles. For this reason, the method is also applicable in sewage systems for wastewater transport [54].

The theoretical level of measurement precision in this method depends on the sensor’s inclination angle *φ*, the signal’s bandwidth, and the width of the segmentation window, along with the associated windowing errors. The function determining the cross-correlation recognizes only the assigned correlation area. In cases where a small number of particles is present in the fluid, measurement accuracy may decrease. In relation to the method using the cross-correlation function, the equation for particle velocity $\;V_{x}\;$ takes the form of Equation (1).

(1) $$V_{x}=\frac{c\tau (x)}{2T_{PR}sin(\varphi )}$$

where: $V_{x}$—particle velocity, τ(x)—delay corresponding to the global maximum of the cross-correlation function at position *x*, *φ*—sensor inclination angle relative to the fluid motion, equal to 45°, *T_PR_*—time between emissions of two pulse sequences, and *c*—sound speed in the propagation medium [19].

A major advantage of this method is its capability for profiling fluids in both open and closed channels. However, in channels that are small relative to the device size, fluid turbulence can significantly affect the accuracy of the measurements. Significant errors also occur in the case of pure water. Another issue is when large objects in the fluid get stuck above the level of the measuring device (e.g., wedging near it), obstructing the transducer and leading to erroneous measurements. In practice, frequent checks of its status are required.

### 3.2. Construction of a Device for Measuring Wastewater Flow in an Open Channel

**Figure 1:**
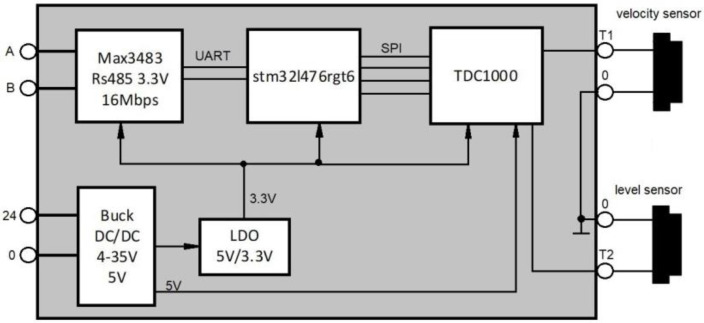
The electrical schematic of the measuring device. Source: own development based on [56].

Figure 1 presents an electrical schematic of the developed and constructed device for measuring flow in an open channel. The device utilizes the specialized Texas Instruments TDC1000 circuit, an ultrasonic front-end (AFE) that allows for hardware generation and analysis of ultrasonic signals with automatic gain profile adjustment, pulse count regulation from 1 to 13, an operating temperature range from −40 to 125 °C, and AEC-Q100 certification. The TDC1000 transducer works in conjunction with a 32-bit ARM Cortex M4 processor with RISC architecture (STM32), which is used for signal analysis, correlation determination, and communication protocol management.

**Figure 2:**
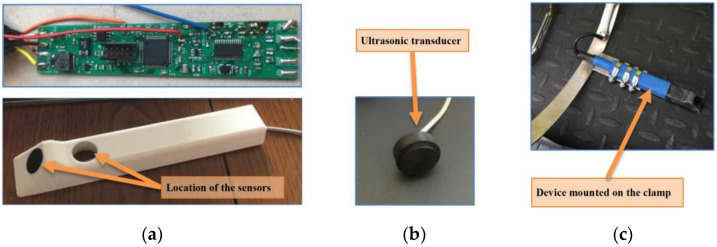
Photos of the electronic circuit (**a top**), digital printout of the device’s casing (**a bottom**), ultrasonic transducer (**b**), and device mounted on the clamp (**c**). Source: own.

Moreover, the system integrates a converter for 5 V power conversion, a 5/3.3 V voltage regulator, and a fast RS485 circuit, enabling communication according to the industrial RS485 standard between the device and the management system, along with two ultrasonic transducers. The first is mounted at a 45° angle for liquid flow measurement, and the second is perpendicular to the surface for measuring liquid height. An accelerometer, gyroscope, and magnetometer are also built into the device, allowing for precise determination of the sensor’s spatial orientation in the fluid. Proper positioning of the device is necessary for accurate measurements. Figure 2 displays images of the constructed measuring device: the electronic circuit, the digital printout, the ultrasonic transducer, and the mounting on the clamp.

**Figure 3:**
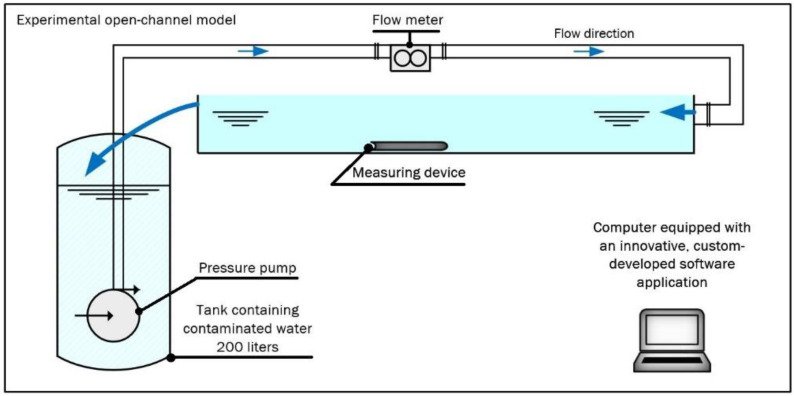
A schematic of the setup for measuring the flow rate in an experimental open channel. Source: own development based on [56].

Figure 3 presents a schematic of a laboratory measurement setup consisting of the measuring device placed in a *ϕ* = 0.15 m diameter half-pipe, supplied with tap water containing particles of crushed pellets, simulating sewage. A submersible pump controlled by a three-phase powered inverter system is used for supply. An Optiflux 1000f electromagnetic flow meter from Krohne is installed in the half-pipe [57]. The experimental setup allowed for the control of the flow rate *Q*_set_ ranging from 0 to 210 L/min.

The principle of measuring speed and liquid level in the channel, showing the propagation of ultrasonic waves and subsequent measurement layers, is as follows: The measuring device is mounted at the bottom of the channel. The device has two sensors: a C_H_ level (depth) sensor and a C_V_ (velocity) sensor. The C_V_ sensor is inclined at a 45° angle to the channel bed. The C_H_ sensor is positioned perpendicular to the channel bed.

The C_V_ and C_H_ sensors are initialized with optimal parameters determined during the research. These parameters include:

- *N*_offset_—the number of initial samples discarded, containing signals reflected from the walls of the C_V_ and C_H_ sensors;
- *δ*—the number of signal samples used to determine the *N*_offset_ parameter;
- *T_PR_*—the time between transmission and reception of successive pulse series emitted by the C_V_ sensor;
- *L_W_*—the width of successive layers, considered within a data frame composed of 2000 signal samples;
- *N*—the number of signal samples considered in the correlation analysis;
- *TH*—threshold value for determining the liquid level by the sensor C_H_.

The data frame corresponding to the signal recorded by the C_V_ sensor consists of 2000 signal samples. The number of initial samples discarded, marked as *N*_offset_, is ignored in the analysis. The subsequent 2000 *N*_offset_ signal samples are subjected to correlation analysis, with the frame divided into layers of width *L_W_*, and only *N* samples from each layer are included in the correlation analysis. Next, the C_H_ sensor emits an ultrasonic signal, and the liquid level *H* is calculated. Using these values, the cross-sectional area *A* of the channel for the successive layers is determined according to the profile calculated in computer simulations, assuming a profile for turbulent flow [58] using Equation (2).

(2) $$A=\frac{1}{2}r^{2}\left(2\cos^{-1}\left(\frac{r-h}{r}\right)-\sin\left(2\cos^{-1}\left(\frac{r-h}{r}\right)\right)\right)$$

where: *A*—the area of a circle with radius *r* (measured independently) and height *h* (determined by the device).

Although water is continuous, the velocity of flow in open channels is not uniform across the entire cross-section. This non-uniform velocity distribution necessitates dividing the flow into spatial measurement layers aligned with the theoretical velocity profile. The number of layers, each of the same length, was determined experimentally during our research to ensure accurate representation of the flow characteristics.

**Figure 4:**
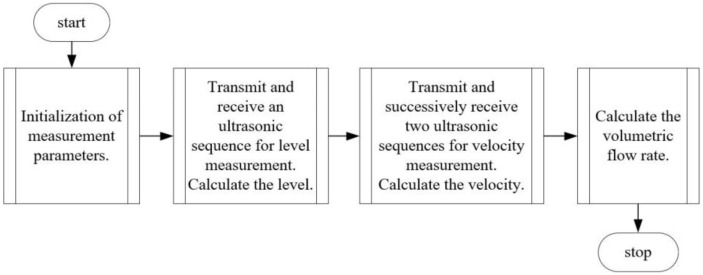
The block diagram of the software implemented in the measuring device. Source: own development.

In the next step, two ultrasonic sequences are transmitted from the C_V_ sensor, and the liquid velocity *v* is calculated. The liquid volume flow *Q*_det_ is calculated as the product of velocity *v* and area *A*. Figure 4 shows the general block diagram of the algorithm implemented in the measuring device. The computational studies conducted under test conditions were carried out in the MATLAB R2021b environment, which provided a robust platform for developing and testing the algorithm. In this environment, various scenarios were simulated to evaluate the algorithm’s performance and accuracy under different flow conditions. Following the successful validation of the algorithm in MATLAB, it was then converted into C language to ensure compatibility with the device’s microcontroller. This conversion involved careful consideration of the algorithm’s structure and functionality to optimize its performance for real-time applications. The implemented algorithm in the microcontroller enables efficient processing and reliable operation of the device in practical settings.

Experimental measurements were performed in several series, with different sensor parameter values (initialization with various values). The subsequent results will refer to these measurement series.

### 3.3. Determination of the Velocity

One of the specific objectives of the research was to perform multivariate analyses of the recorded signals and to select parameter values for accurate velocity measurement based on the results. Therefore, several measurement series were carried out, each time using the same device built as described in the previous section. This section describes the algorithm for calculating flow velocity within a channel and the results of assessing the impact of selected device parameters on the results obtained.

**Figure 5:**
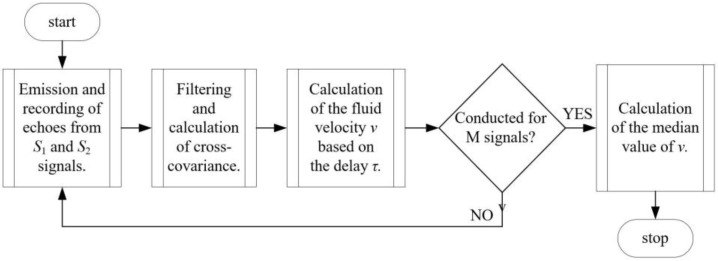
The block diagram of the algorithm for calculating fluid velocity in an open channel. Source: own development.

Signals recorded by the C_V_ velocity sensor were processed according to the algorithm, the block diagram of which is shown in Figure 5. Firstly, after initializing the sensor parameters, two successive ultrasonic signal sequences *S*_1_ and *S*_2_ are emitted and then recorded by the C_V_ sensor. It was assumed that the *S*_1_ and *S*_2_ sequences, consisting of a series of pulses with a base frequency of *f*_0_ = 1 MHz, are emitted by the transmitting device at an interval equal to *T_PR_*. The *S*_1_ wave, after reflecting off a particle flowing in the liquid, is recorded by the receiving transducer with a delay *T*_X1_. It is assumed that the echo of the S_2_ wave, reaching the transducer with a delay *T*_X2_, is reflected off the same particle after it has traveled a distance *d* at a constant velocity *V*_x_. To calculate the fluid velocity *v*, the recorded signals were pre-processed using a band-pass filter. All signal components other than the carrier frequency of the transmitted signal, which was 1 MHz, were filtered out. In the filtration process, a first-order digital elliptical band-pass filter was used, with normalized edge frequency defined within the range of 0.27–0.28. The resulting filter has a passband attenuation value Rp = 1 dB and a stopband attenuation value Rs = 40 dB. In the studies, the cross-covariance function was used. The velocity is determined based on the known *T_PR_* value and the delay corresponding to the global maximum of the cross-correlation function τ, calculated for the two ultrasonic signal sequences *S*_1_ and *S*_2_.

### 3.4. Determination of the Liquid Level

**Figure 6:**
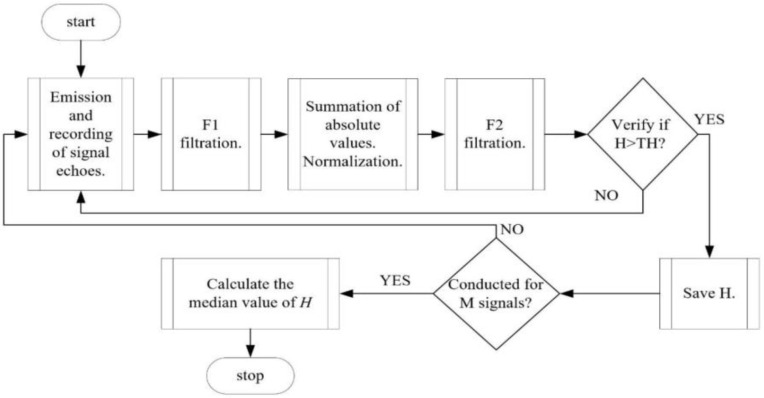
Block diagram of the C_H_ sensor data processing algorithm. Source: own work.

**Figure 7:**
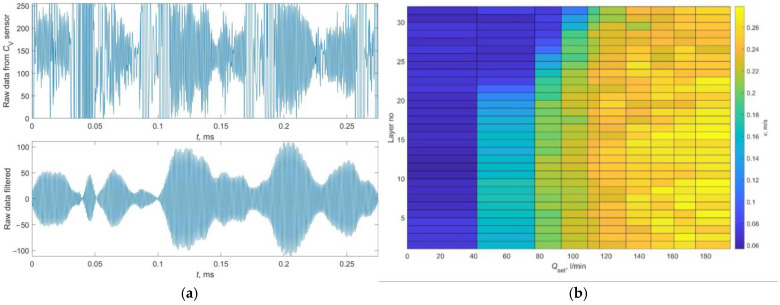
Example raw data gathered from sensor *C*_v_ (**a top**) and raw data after filtration (**a bottom**). Example calculation results of velocity *v* [m/s] depending on *Q*_set_ [L/min] for 32 signal layers, with *T_PR_* = 1 ms, *L_W_* = 32, *N*_offset_ = 350, and *N* = 8 (**b**). Source: own work.

According to the flow calculation procedure described in Figure 4, in addition to the velocity *v* measurement in the designed device, the liquid level *H* was also determined. This section presents the principle of the measurement method and the algorithm for processing signals recorded by the level sensor C_H_. Figure 6 illustrates the block diagram of the algorithm for processing signals to calculate the liquid level *H* in the channel. Firstly, the sensor emits a beam of pulses at the base frequency *f*_0_ = 1 MHz. In this task, it was assumed that the beam, which is emitted perpendicular to the channel bed surface, reflects off the liquid surface and returns to the receiver after some time. Next, the emitted and recorded signal from the C_H_ level sensor is subjected to filtration using a band-pass filter (Filtration F1). The purpose of this process is to filter out all frequency components irrelevant from the perspective of the carrier wave frequency *F* = 1 MHz. In this task, a first-order digital elliptical band-pass filter was used, with normalized frequency defined within the range: [0.27–0.28]. The resulting filter has a passband attenuation value of 1 dB and a stopband attenuation down from the passband value of 40 dB. The idea of the developed algorithm is based on the fact that the signal segment reflected from the surface of the liquid filling the open channel is characterized by a higher amplitude compared to signal segments reflected from particles carried by the liquid. Therefore, it was proposed to sum a series of consecutive modules (absolute values) of signals, assuming that locally large amplitudes of signal segments reflected from particles, will be located in varied locations, whereas the liquid level will be at the same height. A set of 200 recorded signals, obtained in a given measurement session, was divided into four data packets. Then each was separately processed according to the algorithm, the individual stages of which are visualized in Figure 7. Subsequently, the processed signals were subjected to the F2 filtration, in accordance with the developed algorithm. In this task, a first-order digital elliptical band-pass filter with normalized edge frequency set within the range of [0.02–0.06] was applied. The resulting filter has a band-pass attenuation of 0.5 dB and a stop-band attenuation of 20 dB. The purpose of the F2 filtration is to remove rapidly varying frequency components from the signal. Next, the location (sample number) of a sudden amplitude jump, which is assumed to be directly correlated with the signal reflected from the liquid surface is determined for a value of TH, set as a multiple of the standard deviation and considered as a threshold. The median value, determined from successive packets of signals, is considered as the time of arrival of the signal reflected from the surface. After calculation, taking into account the transmission speed, the height *H* [mm] is determined. This process was conducted under laboratory conditions with a known liquid height for a controlled flow rate *Q*_set_ in the range of 0–200 L/min.

## 4. Results

### 4.1. Assessment of the Impact of C_V_ Sensor Parameters on the Results Obtained

Figure 7a presents example waveforms of the raw and filtered data gathered from sensor *C*_v_. To determine the basic operating parameters of the C_V_ sensor, considering a series of technical limitations, the first step was to determine the values of the *N*_offset_ and *T_PR_* parameters. For this purpose, measurement series were carried out for three different *T_PR_* values: {1, 2, 10} ms. Then, using different numbers of signal samples *δ* in the range [50–200] and different *N*_offset_ values in the range [300–900], *τ*_max_ was determined and based on it, the velocity *v*. The experimental setup allowed for controlling the flow *Q*_set_ in the range from 0 to 210 L/min. Based on the analysis of the obtained dependencies, it was found that for *T_PR_* = 2 ms at flows exceeding 150 L/min, the calculated velocities *v* fell below zero, which was caused by the mismatch of the *T_PR_* parameter to the actual velocity of particles present in the liquid. Meanwhile, for *T_PR_* = 10 ms, the calculated *v* values oscillated around 0. For all measurement series conducted, it was evident that regardless of the *δ* and *N*_offset_ values, correct results, i.e., those for which a linear, monotonically increasing form of the *v*(*Q*_set_) curve was obtained, were achieved only for the *T_PR_* = 1 ms parameter. The procedure for calculating covariance for the entire signal sequence increases the processor load built into the designed measuring device, particularly extending the calculation time. Therefore, subsequent studies aimed to select the optimal values for the *N* and *L_W_* parameters, with *T_PR_* = 1 and *N*_offset_ = 350. A preliminary assessment of the impact of *N* and *L_W_* parameters on the results was made based on the analysis of the *v*(*Q*_set_, *LayerNo*) dependencies. Example calculation results for *T_PR_* = 1 ms, *L_W_* = 32, *N*_offset_ = 350, *N* = 8 are shown in Figure 7b. The presented velocity distribution varies across different layers, which is consistent with the theoretical values calculated using the numerical model presented in [56].

Based on the gathered results, we stated that for *N* < 8, the calculated velocities oscillated around 0 m/s, and for *N* > 8, the obtained dependencies were similar. Based on the preliminary assessment of the obtained dependencies, it was concluded that both parameters, *N* and *L_W_*, are essential for the correct estimation of particle velocities in individual layers of an open channel, and their selection should be automated. Therefore, to select the optimal parameter values, an optimization process was conducted using a genetic algorithm, which was automated in the form of a computer script. In this process, the objective function was the minimization of the norm of residuals calculated for the estimated flows *Q*_det_ and the set flows *Q*_set_, where the ideal values are characterized by a linear relationship *Q*_det_ (*Q*_set_). As a result of the optimization process, the following parameters were obtained: *L_W_*_opt_ = 12, *N*_opt_ = 8.

### 4.2. Example Results of the Liquid Level

**Figure 8:**
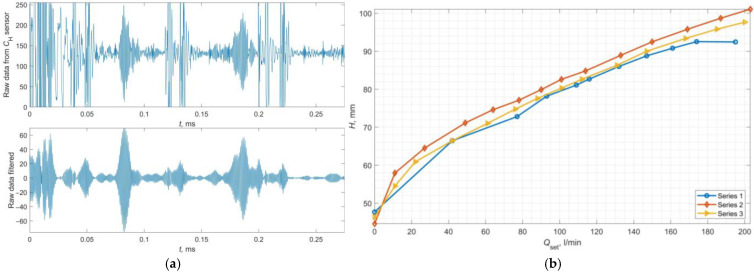
Example raw data gathered from sensor *C*_H_ (**a top**) and raw data after filtration (**a bottom**); the calculated dependency *H*(*Q*_set_) for example measurement series (**b**).

Figure 8a presents example waveforms of the raw and filtered data gathered from sensor *C*_H_. Figure 8b shows the values of *H* calculated for different set flow rates (*Q*_set_) in three example measurement series. Based on the *H*(*Q*_set_) relationships obtained, a monotonic curve character was achieved, reaching around 100 mm for maximum flows. In some series, the calculations for the highest flow value indicated an incorrect (too low) *H* value. For zero flow, the liquid level was 45 mm, directly resulting from the limitations of the developed laboratory measurement system. Furthermore, it was observed that the liquid level rises exponentially with the initial increase in the set flow rate, changing to a linear increase relative to *Q*_set_ after crossing about 30 L/min.

The *TH* value, defined as a multiple of the standard deviation of the first few dozen samples of the filtered signal, is crucial for accurate level measurement and, consequently, for determining the flow rate of liquid in an open channel. Optimal *TH* values were determined empirically by comparing the actual liquid level in the channel with the values calculated by the developed device.

### 4.3. Validation under Laboratory Conditions

An important part of verifying whether the developed device correctly performs flow measurement is its validation. The validation studies included an analysis of the linearity of the functional relationship *Q*_det_ = *f*(*Q*_set_) and an analysis of the repeatability and uncertainty of measurement. The research was conducted using four instances of the device built according to the procedure described. The velocity and level sensors were initialized with parameter values determined within the analyses.

#### 4.3.1. Repeatability Analysis

**Figure 9:**
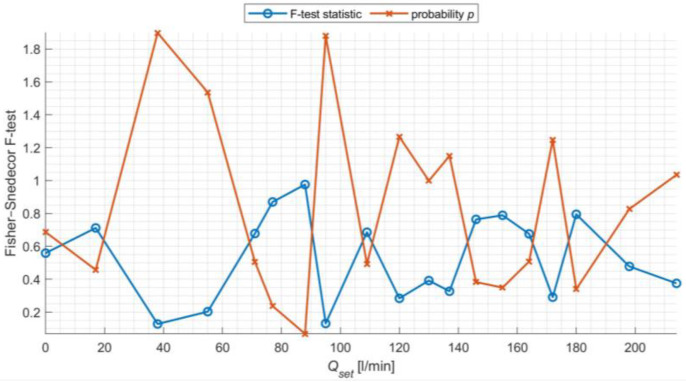
F-test statistic values with probability *p* for successive set flow sizes *Q*_set_.

Initially, an analysis of the repeatability of measurements was performed in four series labeled S1–S4, with 250 recordings of the output variable *Q*_det_ for given flow rates *Q*_set_ ranging from 0 to 210 L/min, using a single device instance, installed under the same conditions in the same laboratory setup. For this purpose, a one-way ANOVA was used for each of the tested *Q*_set_ flows. The null hypothesis assumed that the mean values of *Q*_det_ recorded in the four series of 250 measurements each are the same. The alternative hypothesis, defined as the opposite, states that at least one series significantly differs from the others. One-way ANOVA requires the assumption that the samples taken for analysis are independent, drawn as simple random samples, and belong to a set with a normal distribution with equal variances. Having met the first three conditions, for each of the four measurement series S1–S4, a test for belonging to a population with a normal distribution was conducted using the parametric Z-test, assuming a known mean and standard deviation calculated for each of the four series. The study assumed that the null hypothesis confirms the tested measurement series belonging to a set with a normal distribution with the given mean, and the alternative hypothesis defines that the tested measurement series does not belong to a normal distribution with the given mean. For all four measurement series S1–S4, for each *Q*_set_ flow, the null hypothesis was confirmed at a 5% significance level. In the ANOVA analysis, the Fisher–Snedecor F-test value is calculated, which is then tested for belonging to the critical area $CA=\left\{F:F\geq F_{\alpha }\right\},$ determined from the distribution for (*r* − 1, *n* − *r*) degrees of freedom. In this study, the critical value $F_{\alpha }=2.614$, where *r* = 4, *n* = 1000. F-test statistics values, calculated for all flows, are compiled with *p*-values in Figure 9. All calculated statistics do not belong to the critical area, indicating no basis for rejecting the null hypothesis and concluding that the tested means are homogeneous.

#### 4.3.2. Uncertainty Analysis

To determine the reliability of flow measurements recorded by the developed device, an uncertainty analysis was performed. The calculations used 250 values of the variables *V* and *H* recorded by a single device instance in four measurement series S1–S4 and the size of the variable R, which was determined in an independent measurement with a caliper at *R* = (0.070 ± 0.001) m and was constant. The output quantity *Q* is calculated based on the values of the level of channel filling *H*, velocity of the sewage V in a circular channel of radius *R*, using the functional relationship $\;Q=f\left(V,R,H\right)$. In the task at hand, the developed device was used to record three input variables: radius *R*, height *H*, and velocity *V*, based on which the output variable, the flow quantity *Q*, was calculated. Therefore, standard uncertainties of type A for the input variables $u\left(h\right),\;u\left(v\right)$ were estimated. The standard uncertainty u(r) was estimated using Type A method at the level of 1 mm. Additionally, the examination of the correlation coefficient between the output variables *H* and *V*, calculated using Pearson, Kendall, and Spearman methods, showed no such relationship. For all tested flows, this coefficient was at most 0.04. Due to the lack of correlation, it was assumed that the combined standard uncertainty $u_{c}\left(q\right)$ could be calculated as the positive square root of the combined variance (3).

(3) $$u_{c}^{2}\left(q\right)={\left(\frac{\partial q}{\partial h}\right)}^{2}u_{c}^{2}\left(h\right)+{\left(\frac{\partial q}{\partial v}\right)}^{2}u_{c}^{2}\left(v\right)+{\left(\frac{\partial q}{\partial r}\right)}^{2}u_{c}^{2}\left(r\right),$$

where: $\frac{\partial q}{\partial h},\frac{\partial q}{\partial r}\;i\;\frac{\partial q}{\partial v}$ are partial derivatives, known as sensitivity coefficients.

**Figure 10:**
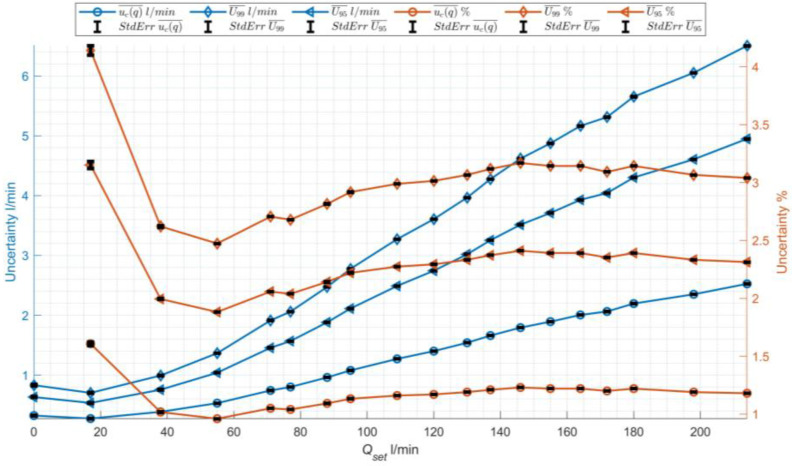
Left axis: values of $\bar{u_{c}\left(q\right)}$ calculated as arithmetic averages for the four measurement series S1–S4, with error bars representing the standard error, and average extended uncertainties $\bar{U}$ in L/min, calculated with 95 and 99, also with error bars for the standard error. Right axis: percentage values.

Figure 10 summarizes the calculated values of combined standard uncertainties *u*_c_(*q*_S1_)–*u*_c_(*q*_S4_) for the four measurement series S1–S4 for each set flow rate *Q*_set_ as their arithmetic mean $\bar{u_{c}\left(q\right)}\;$ and the arithmetic mean of the extended standard uncertainties *U* for different components of the combined uncertainty at two confidence levels: 95% (*k* = 1.96) and 99% (*k* = 2.576). The curve for the 99% confidence level is higher, reflecting greater uncertainty at the higher confidence level. Figure 10 also shows the corresponding percentage values relative to the set flow rate, marked in red on the graph. The horizontal axis represents the measured flow rate *Q*_det_ in L/min; the left vertical axis indicates the average combined standard uncertainty and the average extended uncertainty, both expressed in L/min, while the right vertical axis shows the uncertainties as percentages (%).

The average combined standard uncertainty in L/min increases linearly with the flow rate, rising from approximately 0 to 2.5 L/min. Similarly, the extended standard uncertainty increases, though it starts at around 1 L/min and exceeds 6 L/min at very high flow rates. The extended uncertainty in percentage follows a similar trend to the combined uncertainty. For low flow rates, the percentage uncertainties decrease sharply—from over 6% to around 3% for the extended uncertainty at 99% (*U*_99_), and from over 1.5% to about 0.5% for the combined standard uncertainty. After a slight rise, these values remain stable for higher flow rates.

Although the uncertainty values expressed in L/min (blue lines) increase noticeably with the flow rate, the corresponding percentage uncertainties (red lines) remain relatively low. This suggests that for high flow rates, even though the absolute uncertainty in L/min increases, its relative impact in percentage terms becomes less significant and can be considered acceptable in the context of the measurements.

The plots also display the standard error magnitude for each curve using black whiskers. All errors are negligible and nearly invisible on the charts, indicating that the uncertainties across different measurement series are very similar. Additionally, a Wilcoxon signed-rank test was conducted, which showed with the highest probability that the uncertainties for the respective flow rates originate from the same distribution (as evidenced by the consistent *p* = 1 value). The obtained measurement uncertainties were deemed satisfactory.

#### 4.3.3. Analysis of the Relationship between the Set and the Determined Flow Rate

**Figure 11:**
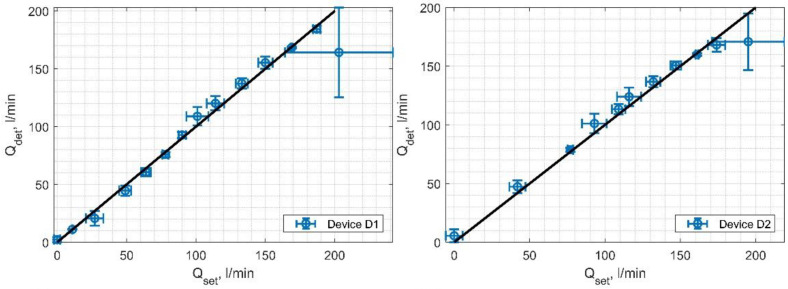

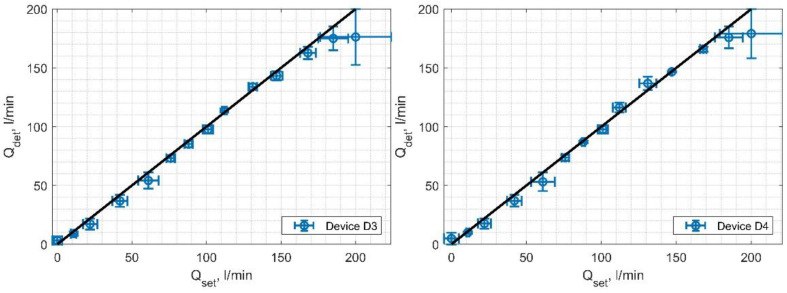
Average *Q*_det_ values as a function of *Q*_set_ along with the linear dependency curve for four device specimens.

Subsequently, a study of the linearity of the relationship between the determined flow and the set flow was conducted. For this purpose, four measurement series were performed, one series for each device instance, for set flow rates *Q*_set_ ranging from 0 to 210 dm³/min. For each set flow rate *Q*_set_, within one series, 250 measurements of the flow *Q*_det_ were made, calculated according to the procedures defined during the research. The values of the norm of residuals were calculated for each device instance. On average, this error amounted to 31.85 L/min. The values of the coefficients of determination were also calculated for fitting the *Q*_det_(*Q*_set_) curves to an ideally linear relationship. On average, this coefficient was 0.983. The discussed results are visualized in Figure 11. It was further determined that for flows in the range of 40–190 dm³/min, the developed device performs the measurement of the liquid volume flow with an average inaccuracy of 5.49%. In the range of low and very high speeds, adjustment of the device parameters is necessary, related to the number of pulses emitted within the sequences *S*_1_ and *S*_2_, and the *T_PR_* time.

## 5. Discussion

Our research led to the optimization of parameters to achieve a linear relationship *Q*_det_(*Q*_set_) = *Q*_set_, considering hardware limitations, energy consumption, and calculation time. Measurement data were proven to be repeatable using ANOVA, with uncertainties remaining within a satisfactory range. The combined standard uncertainty increases linearly with the increase in flow rate. However, it does not exceed 1.22% of *Q*_set_, with the median value being 1.18% of *Q*_set_. The extended standard uncertainty does not exceed 4.83% of *Q*_set_, with the average value being 3.51% of *Q*_set_ and not exceeding an average of 3.47% of *Q*_set_ for flows above 40 L/min. Therefore, the device’s operation was validated under steady, unchanging flow conditions.

Furthermore, an average inaccuracy of 5.49% was observed, with room for further improvement at extreme low and high flow rates. These improvements could be achieved by adjusting device parameters, such as the number of pulses emitted in the *S*_1_ and *S*_2_ sequences and the *T_PR_* time. Following the design and testing phases, a series of measurement devices were constructed and deployed in accordance with predefined standards. These devices were strategically placed at the base of eight sewer manholes for two weeks to conduct a detailed measurement campaign. The data obtained from this initiative showed a strong correlation with the sewage discharge volumes reported by the water utility. Similar measurement exercises are ongoing in other sanitary systems operating under comparable conditions. The devices developed through this project are proving invaluable for measurement campaigns aimed at collecting comprehensive data on the operational dynamics of sanitary networks. This data is crucial for the development and calibration of simulation models that accurately represent these systems.

## 6. Conclusions

The growing demand for efficient wastewater management and the need for cost-effective monitoring solutions highlight the importance of accurate flow measurement devices. This research provides a significant advancement in the field, offering a reliable and affordable option for measuring wastewater flow rates, which is crucial for optimizing management practices and ensuring compliance with environmental standards. Traditional methods often involve significant expenses and limitations, making it challenging for many facilities to maintain compliance and optimize operations. The growing demand for efficient wastewater management and the need for cost-effective monitoring solutions highlight the importance of accurate flow measurement devices. This paper presents the results of research aimed at developing a low-cost device for measuring wastewater flow in open channels, designed to minimize interference with the natural flow, particularly for open channels used in sewage systems with diameters of approximately 50 cm. In a laboratory setup, consisting of a cylindrical half-pipe, flows of contaminated water at varying rates—up to 210 L/min—were tested, with the measuring device installed at the bottom of the half-pipe.

A key benefit of this method is its ability to profile fluids in both open and closed channels. However, in channels that are relatively small compared to the size of the device, fluid turbulence can greatly impact measurement accuracy. Additionally, substantial errors can arise when measuring pure water. Another concern is the potential for large objects in the fluid to become lodged above the level of the measuring device (for instance, by wedging near it), which can obstruct the transducer and result in inaccurate readings. Consequently, regular checks of the device’s status are necessary in practical applications. The developed method demonstrates higher percentage measurement uncertainties for very low flow rates, exceeding 4%; however, uncertainties significantly decrease for larger flow rates. Nevertheless, the linearity of the relationship between the calculated flow and the set flow yields unsatisfactory results for flow rates above 200 l/min; thus, we do not recommend its application in such cases

## Data Availability

The raw measurement data can be made available upon request by contacting the authors directly.
